# Malaria serology for surveillance, insights into immunity, and vaccine development

**DOI:** 10.1128/iai.00031-26

**Published:** 2026-07-13

**Authors:** Pailene S. Lim, Jhobert Bernal, Janani Karunarathne, Rhea J. Longley

**Affiliations:** 1 Walter and Eliza Hall Institute of Medical Research 549398 , Parkville, Victoria, Australia; 2 Department of Medical Biology, University of Melbourne 2281, Parkville, Victoria, Australia; 3 Mahidol Vivax Research Unit, Faculty of Tropical Medicine, Mahidol University, Bangkok, Thailand; University of California Davis, Davis, California, USA

**Keywords:** malaria, surveillance, immunology, *Plasmodium vivax*, luminex, memory B cells, functional antibodies, sero-surveillance, serological testing and treatment

## Abstract

Malaria remains an infectious disease of global health concern. Recent approval and implementation of two vaccines for use for prevention of *Plasmodium falciparum* malaria in children in the African region is predicted to significantly reduce the burden of this disease. However, efforts are still required to improve efficacy and durability of these vaccines in target populations, and many other challenges remain to achieve malaria eradication. These include emergence and predominance of non-falciparum malaria in countries nearing elimination of *P. falciparum*, resurgences in malaria-free settings, and impacts of changing populations and ecology on transmission. Novel tools are needed to target neglected *Plasmodium* species, notably *Plasmodium vivax,* the second most common cause of human malaria. *Plasmodium* infections induce robust immune responses, meaning *Plasmodium-*specific antibodies can be used as both surveillance tools and as markers of immunity to clinical disease. In this mini-review, we discuss these use cases of malaria serology. We detail the concept behind serological surveillance and the need to understand antibody longevity, and discuss the application of this approach in endemic countries for both improved surveillance and as an intervention, with a focus on *P. vivax* while touching on other neglected *Plasmodium* species. People living in malaria-endemic areas naturally gain clinical immunity to malaria, meaning sero-epidemiological studies can also be used to understand the targets and functions of protective immunity. We review new studies on protective immunity, covering research on both *P. falciparum* and *P. vivax*, and highlight how these findings can be leveraged to maximize existing and novel vaccine design.

## INTRODUCTION

Malaria, caused by *Plasmodium* parasites and transmitted by *Anopheles* mosquitoes, remains a major public health problem, causing an estimated 282 million cases per year in 2024 (1). Substantial challenges to achieving the ambitious World Health Organization (WHO) goal of malaria elimination persist. In the Asia-Pacific and Americas, the major threat relates to the high proportion of cases (up to 63%) caused by *Plasmodium vivax* (1), a parasite species with relapsing infections caused by a liver-stage hypnozoite reservoir (2). Additionally, the Asia-Pacific faces the emerging threat of *Plasmodium knowlesi*, a zoonotic malaria that can cause severe and fatal infections. Despite nearly all endemic countries in the Asia-Pacific and Americas committing to eliminating malaria by 2030 (3), realizing this aim requires the development of innovative new tools to specifically target these historically neglected *Plasmodium* species. Importantly, both licensed malaria vaccines (RTS,S and R21) provide protection against *Plasmodium falciparum* and are targeted at children in Sub-Saharan Africa; they are not designed for use against these neglected *Plasmodium* species that are predominant in the Asia-Pacific and the Americas.

**Fig 1:**
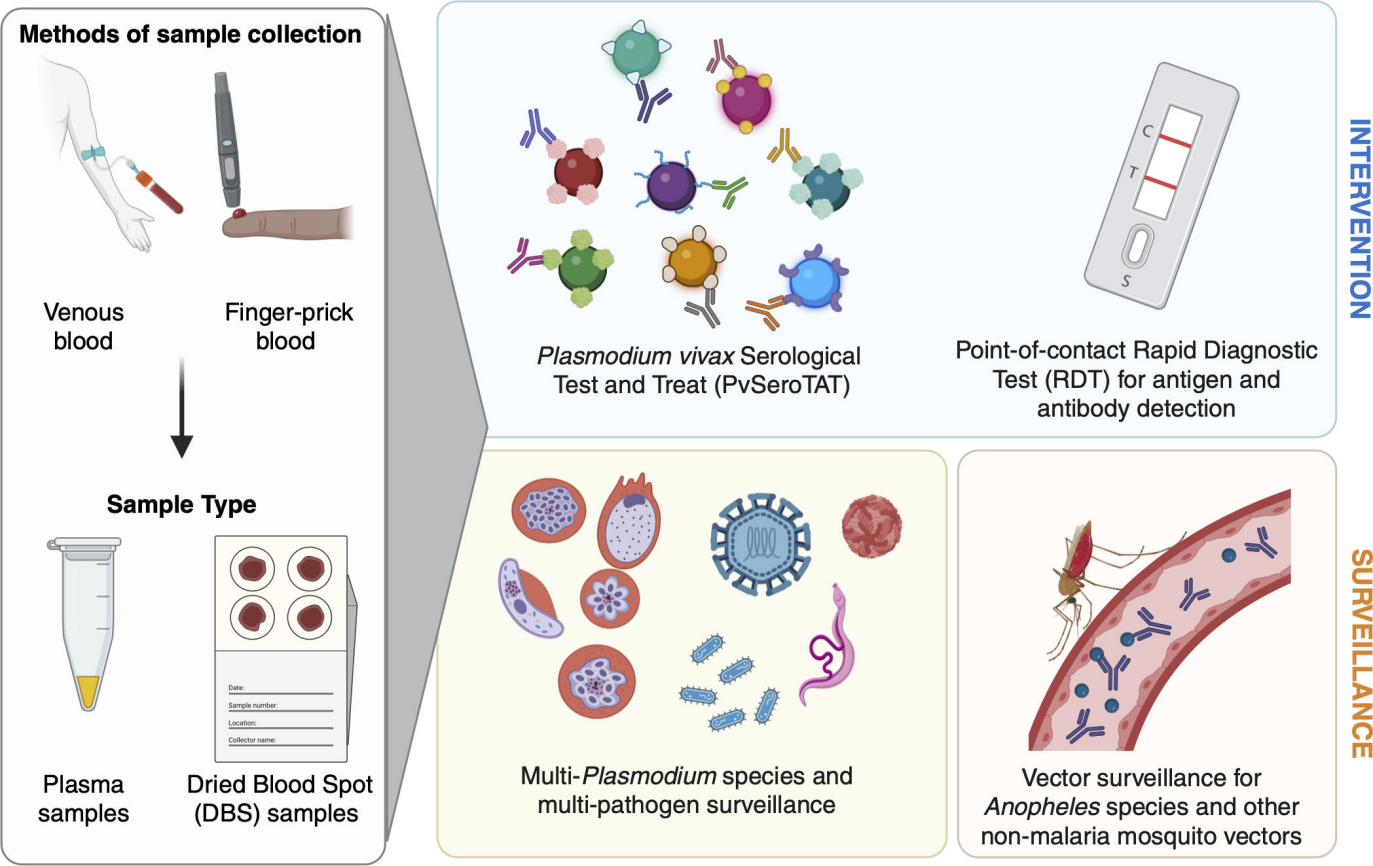
Innovative serological assays as a tool for malaria intervention and surveillance of infectious diseases. Left panel: Small amounts of plasma isolated from venous blood/finger prick blood samples or extracted from dried blood spot samples can be used to perform various serological assays. Top panel: Recent *Plasmodium vivax* infection (<9 months post initial blood-stage infection) and likely hypnozoite carriage can be detected using a panel of serological exposure markers, which allows for targeted testing and treatment (PvSeroTAT). Point-of-contact rapid diagnostic tests (POC RDTs) are also under development to aid use of PvSeroTAT as a *P. vivax* malaria intervention. Bottom panel: Serology can also be used to develop multi-*Plasmodium* species and multi-pathogen antibody assays, and for vector surveillance. Detection of human antibodies to *Anopheles* species-specific antigens can be used to track human-vector contact. These pathogen- and vector-level serological applications can be integrated to improve malaria and other infectious disease surveillance and guide multi-disease elimination. This figure was created with BioRender.

**Fig 2:**
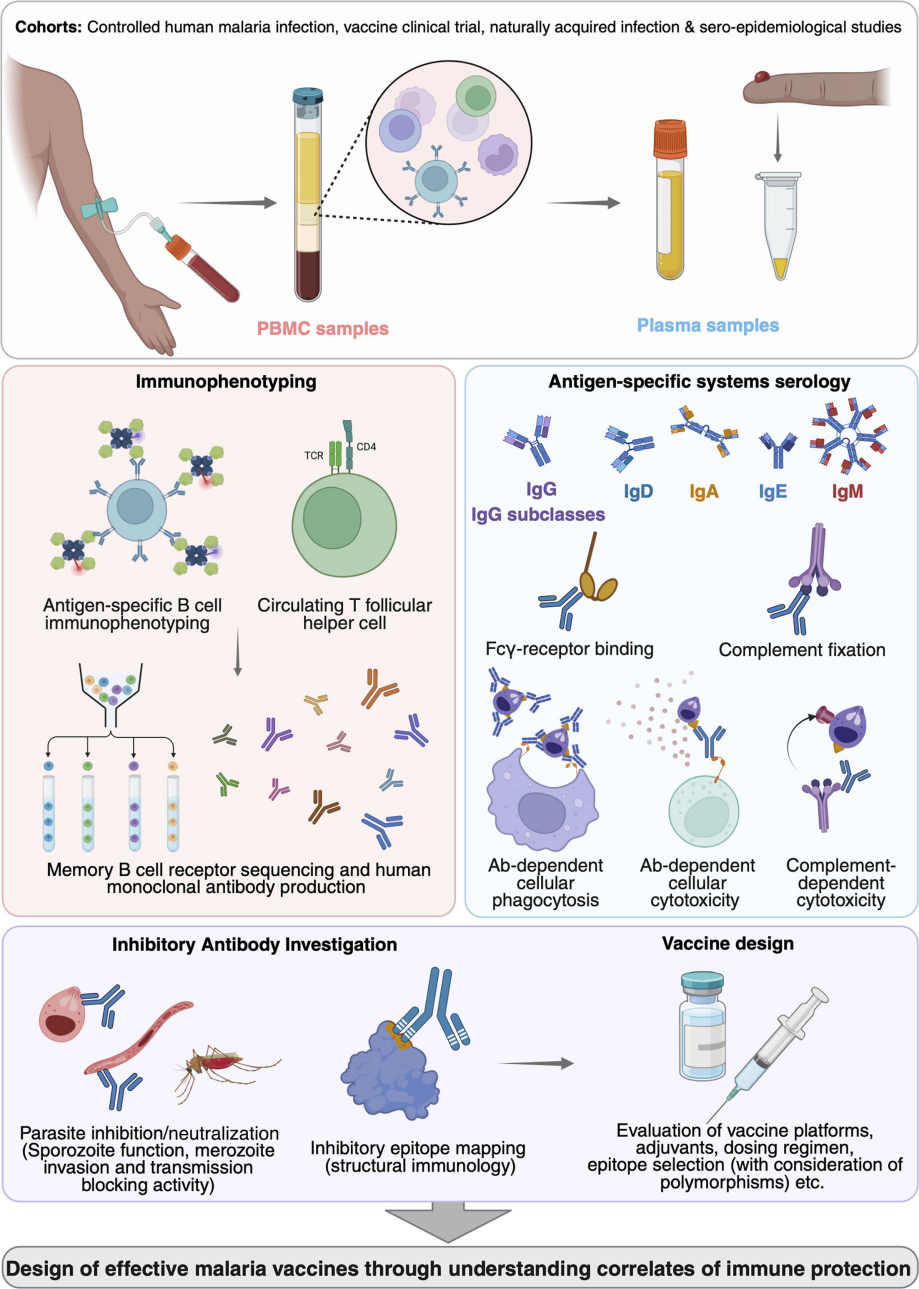
Understanding correlates of immune protection against *Plasmodium vivax* to design effective malaria vaccines. Top panel: Venous blood collected from various cohort studies (i.e., controlled human malaria infection [CHMI] studies, vaccine clinical trials, individuals with naturally acquired infections, and sero-epidemiological studies) can be processed to isolate peripheral blood mononuclear cell (PBMC) samples and plasma samples. Left panel: PBMCs can be used to perform flow-cytometry-based immunophenotyping assays to assess cellular immune responses (notably antigen-specific B cell and circulating T follicular helper cell responses). Antigen-specific memory B cells can further undergo single-cell sorting using tetramer probes to isolate and express human monoclonal antibodies (mAbs). Right panel: Using plasma, a systems serology approach can be utilized to determine if antigen-specific antibody isotypes, IgG subclasses, and functional antibody responses (e.g., Fcγ-receptor binding and complement fixation) are associated with protection against clinical disease. Further cellular-based assays can be used to investigate mechanisms of Ab protection such as Ab-dependent cellular phagocytosis (ADCP) with macrophages/monocytes, Ab-dependent cellular cytotoxicity (ADCC) with natural killer cells, and complement-dependent cytotoxicity (CDC). Bottom panel: Combined, plasma containing antigen-specific Abs and recombinant human mAbs can be investigated for their ability to inhibit or neutralize parasite function across pre-erythrocytic, asexual, and sexual/transmission stages. Epitope mapping of inhibitory and non-inhibitory Abs can elucidate ideal regions of antigens to include in vaccines. Combined with evaluation of vaccine characteristics (e.g., vaccine platforms, adjuvants, dosing regimen, and antigen sequence diversity), these studies allow for the design of effective and robust malaria vaccines. This figure was created with BioRender.

Effective surveillance is the most critical step in the final stages of elimination; otherwise, transmission can rebound with devastating consequences. Unfortunately, settings poised for elimination are also the most challenging for surveillance due to the inefficiency of detecting parasites in blood samples due to low case numbers (4). *Plasmodium* infections induce robust immune responses, meaning *Plasmodium*-specific antibodies can be powerful surveillance tools for both active infection and as markers of exposure (5) and immunity to clinical disease (6). In this mini-review, we highlight key contributions by our group and others in this space, from using antibodies as markers of hidden hypnozoite infections (Fig. 1) to utilizing sero-epidemiologic studies to uncover the targets and mechanisms of protective immunity to guide the design of more effective vaccines (Fig. 2). We use examples from other infectious disease fields to explore where studies of malaria serology could expand in the future, with a focus on generating data that can inform malaria surveillance, programmatic decision-making, and vaccine evaluation in near real-time or on operationally relevant timescales. Critical to generating actionable data is an understanding of antibody temporal longevity following induction, and how this may differ across antibody subtypes.

## UNDERSTANDING ANTIBODY LONGEVITY TO INFORM UTILITY OF SEROLOGICAL SURVEILLANCE AND DESIGN OF DURABLE VACCINES

Serological surveillance has undergone a sharp increase in popularity, particularly following the COVID-19 pandemic, but largely due to its utility in the infectious diseases space where multiplexed technology can enable surveillance of whole groups of pathogens using a single sample (7). Serological surveillance can become actionable when applied to specific settings and research questions, and when important factors such as longevity of antibodies are known. In the context of malaria, we hypothesized that serological biomarkers could be designed and utilized to specifically inform on recent *P. vivax* exposure in the prior 9 months (5). To enable this, we first needed to understand *P. vivax* antigen-specific antibody temporal kinetics. Total IgG antibody responses generally peak 1–2 weeks post-acute *Plasmodium* infection (8), and then wane at differing rates—a factor we demonstrated is at least partially dependent on the specific antigen targeted (5). We were able to demonstrate similar patterns of antibody temporal longevity when measuring IgG1, but surprisingly also evidence of long-lived IgM responses to some *P. vivax* antigens (8). Long-lived IgM has also been detected by others in the context of *P. falciparum* malaria (9) and to a range of other infectious diseases in mice (10). Thus, when we tested the ability of IgM responses to the same panel of *P. vivax* antigens as we did for total IgG, to inform specifically on recent *P. vivax* exposure, we found poorer comparative performance (11). We currently utilize a panel of eight *P. vivax* antigens where total IgG antibody responses can specifically inform on recent exposure to blood-stage *P. vivax* infections in the prior 9 months with 80% sensitivity and 80% specificity (5), in areas of low transmission. In higher transmission settings, due to greater recent and lifetime exposure, total IgG antibody responses to the same *P. vivax* antigens last longer in circulation (12, 13), and this can result in poorer performance of the serological markers in these settings (13, 14). Ultimately, even when considering other antibody biomarkers, such as IgG1, IgG3, and IgM, total IgG responses were still able to better classify recent *P. vivax* exposure in moderate to high transmission settings (13). Importantly, beyond simply measuring antibody levels and their decline over time, they can be integrated into within-host mathematical models, as recently reviewed (15), that can consider and adjust for individual-level factors such as age.

For other infectious diseases such as SARS-CoV-2 (the causative agent responsible for the COVID-19 pandemic), IgA responses have been evaluated as markers of very recent exposure (within the prior month) due to their short temporal longevity (16, 17). Most IgA is found as a secreted dimer in mucosal tissues; however, monomeric IgA is also found within serum and is the second most abundant immunoglobulin following IgG (18). Further, some dimeric IgA can also be found within serum, and is exceptionally short-lasting, and this again has been exploited to develop diagnostic tests for both SARS-CoV-2 (19) and measles (20). With respect to malaria, IgA has been much less studied (21). *Plasmodium* parasites express more than 5,000 proteins, compared with ~10–30 for most viruses (though some do express hundreds [22]), making the study of antigen-specific antibody responses complex. Total IgA responses in serum to *P. falciparum* schizont lysate were found to increase with age, suggesting that they can be markers of cumulative exposure in a high-transmission setting in Ghana (23). In a separate study in Angola covering both high and low-moderate transmission, total IgA responses to a panel of 32 *P. falciparum* antigens revealed evidence of boosting responses to nine antigens in participants who had prior malaria episodes (24). Specifically, IgA responses to the antigens apical membrane antigen 1 (AMA1), reticulocyte-binding protein homolog 2 (Rh2_2030), and heat-shock protein 40 (HSP40) provided the strongest markers for previous *P. falciparum* exposure. We are not aware of any studies on IgA responses following infection with *P. vivax*, and this is thus a key area for further study both as a marker of exposure and potentially protection. Protective IgA responses in relation to *P. falciparum* are discussed in a latter section.

Understanding antibody longevity and the factors driving durable immune responses is not only important for serological surveillance but may also provide insights into how to induce long-lived antibodies through vaccine design. For example, from our observational cohort study in a low malaria transmission area in western Thailand, antibody kinetics over 9 months were monitored to 52 *P. vivax* antigens from 34 symptomatic *P. vivax*-infected individuals (8). We identified that total IgG responses were generally long-lived following natural *P. vivax* infection, at least above a non-malaria-exposed background. However, distinct differences in antibody longevity over 9 months post-acute *P. vivax* infection were clear depending on the antigen, and antigens could be differentiated into inducing either “long-lived” or “short-lived” responses. Investigations into antigenic features revealed that antigens containing a *Plasmodium* export element (PEXEL) motif were more likely to have a longer antibody half-life, whereas antigens with transmembrane domains had shorter IgG1 antibody-secreting cell half-life (8). This potentially could be due to PEXEL motif-containing antigens being exported to the red blood cell (RBC) surface (25–28), thus increasing antigen exposure and uptake by antigen-presenting cells. Protective immunity to malaria is largely driven by neutralizing antibodies (nAbs) (29), meaning immune memory would require the activation and survival of long-lived plasma cells (LLPCs) and memory B cells in the blood and tissues (30–32). For *P. vivax* specifically, these large cohort studies and antigen-specific antibody kinetics have been performed via serology, which limits the insights we can obtain into the cellular drivers of long-lived, potentially protective, antibody responses. Antigen-specific LLPCs are difficult to study in humans as they reside largely in the bone marrow (30) and have limited B-cell receptor (BCR) expression (33); however, memory B cells are able to be identified and characterized within peripheral blood mononuclear cell (PBMC) samples (31, 34) and may shed light on characteristics of the induced immune response (Fig. 2).

There are multiple approaches available for quantifying and/or characterizing antigen-specific B cells, namely enzyme-linked immunospot (ELISpot) or flow cytometry. B cell ELISPOTs are extremely sensitive and can quantify rare memory B cell populations; however, they do not enable phenotyping of B cells (35), whereas flow cytometry can. Phenotyping antigen-specific B cells via flow cytometry relies on fluorescently labeling the antigen of interest, commonly done by expressing an Avi-tagged antigen which is biotinylated, then mixed with a fluorochrome-labeled streptavidin to form a B cell tetramer (36). Streptavidin-specific B cells often contaminate staining, but dual labeling techniques (with two fluorescently labeled streptavidins) can overcome this through identification of double-positive antigen-specific B cells. The use of high-dimensional, multi-parameter spectral flow cytometry (37, 38) can enable deep immunophenotyping of multiple rare antigen-specific B cells in one sample (39, 40). Downstream single-cell sorting of antigen-specific B cells can permit sequencing of BCRs for monoclonal antibody discovery (Fig. 2), which has enabled structure-based vaccine design both for malaria (41) and other infectious diseases (42). Other approaches for high-dimensional single-cell analysis include mass cytometry (43, 44), but downstream analysis such as single-cell sorting and sequencing cannot be performed afterward. Another recent methodological breakthrough has been the incorporation of barcoded oligonucleotide-conjugated antibodies or streptavidin with spectral flow cytometry to integrate multi-omic techniques, such as single-cell RNA sequencing, to investigate protein and gene expression profiles of antigen-specific B cells (45–47). These techniques could be used to help identify if certain memory B cell subtypes, transcription factors, or human germline encoding IgG clonotypes may be associated with long- or short-lived antigen-specific antibody responses. In addition to characterizing antigen-specific memory B cells to better understand antibody longevity, T follicular helper cells (Tfhs) are also important for memory B cell formation and maintenance (31). However, Tfhs remain within lymphoid organs or the spleen and are thus difficult to determine with PBMC samples, although in humans, there are circulating Tfhs that resemble germinal center Tfhs (48), and circulating Tfhs have been associated with protective antibody responses in controlled human malaria infection (CHMI) models (49). Recent work conducting deep sampling on T cells from both blood and tonsils found, in contrast to other work (50), that Tfhs were a tonsil-restricted phenotype but with significant clonal sharing with non-Tfh phenotypes in blood (51). This highlights the potential adaptability and plasticity of T cells in response to their location and in response to infection/antigen exposure, and suggests the importance of tissue-based studies where possible. Tonsil organoid models may therefore be useful to provide insights into antigen-specific B cell development within secondary lymphoid organs (52, 53), but further validation is necessary to determine whether the model can be used for *de novo* adaptive immune responses (54).

## FROM SEROLOGICAL SURVEILLANCE TO CAMPAIGN-STYLE INTERVENTIONS

We have utilized our knowledge of antibody temporal longevity following *P. vivax* infections to build new tools for *P. vivax* serological surveillance. Serological surveillance has a particular use case with respect to *P. vivax,* as *P. vivax* has several distinct life-cycle features compared with *P. falciparum*. Important features that impact our diagnostic capabilities are the presence of hidden or cryptic life-cycle stages both in the liver, such as arrested liver-stage hypnozoites (2), and in the blood-stage with reservoirs of cryptic parasites in the spleen and bone marrow (55, 56), as we have recently reviewed (7). *P. falciparum* does not have liver-stage hypnozoites, and most blood-stage parasites are in the peripheral circulation. Current diagnostic tools rely on taking peripheral blood samples and measuring either the presence of parasites in RBCs directly (microscopy, PCR) or circulating parasite antigen (rapid diagnostic tests [RDTs]). These tools have limited sensitivity for detecting the lower circulating parasite loads common in *P. vivax* (due to the accumulation of blood-stage parasites in the spleen) and cannot detect hidden liver-stage hypnozoites. This has important ramifications both for surveillance efforts and for targeted interventions. Liver-stage hypnozoites form at the time of a primary *P. vivax* infection but do not move into the blood stage of the life cycle, instead remaining “dormant” for many months, to possibly years, before being reactivated by currently unknown signals and rejoining the lifecycle contributing to a relapse of *P. vivax* infection and sometimes disease. Due to the timing of hypnozoite relapse after a primary infection, with most expected to occur within 9 months (57) (even considering staggered re-activation [58]), we hypothesized that our markers of recent blood-stage *P. vivax* infection in the prior 9 months would also be markers of individuals likely to be carrying hidden hypnozoites. This hypothesis could only be directly tested in a context where individuals have no ongoing exposure to infectious mosquitoes, to ensure any detected *P. vivax* infections are due to hypnozoite relapse and not new infections. With collaborators who have designed a unique returning soldier cohort model in Indonesia (59), we were able to demonstrate similar performance of our panel of 8 *P. vivax* biomarkers for detecting individuals who go on to have a *P. vivax* relapse (60). To further evaluate the performance of these *P. vivax* serological markers, analysis of regional genetic variation has demonstrated that several *P. vivax* antigens exhibit high sequence conservation (e.g., 99% identity for the antigen S16), while others show substantial differences in immunogenicity across geographic areas and antigen construct, such as reticulocyte-binding protein 2a (RBP2a) and the Duffy-binding protein region II (DBPII) (61). These findings underscore the critical role of antigenic diversity in the design of effective serological markers for malaria sero-surveillance, while demonstrating adaptation of the current panel to a suitable form for use in vivax-endemic areas globally. The 8-antigen *P. vivax* serological panel has now been used in diverse settings, including in Thailand (5, 62), Brazil (5), Solomon Islands (5), Peru (14), Cambodia (63), and Indonesia (64). This program of work has contributed to the WHO releasing the first preferred product characteristics for a test to detect individuals at risk of relapse (65), with use cases including both surveillance approaches and targeted interventions, as discussed further below.

Disease monitoring and surveillance are essential for identifying health trends and detecting outbreaks. Information generated through these systems enables health agencies to shape policies and decisions that improve community health outcomes, such as where to target their limited resources—this is particularly important in settings where transmission is low or highly heterogeneous (including for identification of hot spots of transmission at small geographic units). Surveillance data also plays a critical role in evaluating the effectiveness and impact of disease control programs. Malaria surveillance employing serological approaches has been widely implemented in multiple endemic countries to characterize individual (66) and population-level exposure (67), as well as transmission intensity (68), though largely in academic research settings. There are a number of challenges for real-world implementation, as have recently been reviewed within India’s country-specific context (69). Our recent efforts to develop *P. viva*x-specific serological markers aim to establish a more refined and standardized framework for assessing *P. vivax* exposure over a defined timeframe (4), and benefits from an embedded Random Forest classification algorithm that provides a simplified binary output of recently exposed in the prior 9 months (or not). As mentioned, not only does this timeframe indirectly inform on hypnozoite exposure but also provides an actionable surveillance timeframe. For example, serological markers can be used for differentiating between historical and more recent exposure. In the Philippines, multiplex serology targeting total IgG antibodies against both *P. falciparum* and *P. vivax* antigens was employed to evaluate three provinces representing distinct malaria transmission profiles (70). Analysis of seroprevalence patterns and antigen-specific antibody responses revealed no evidence of recent *Plasmodium* exposure in areas undergoing elimination or classified as malaria-free, supporting epidemiological data indicating the interruption of local transmission. In contrast, markedly elevated antibody levels were observed among individuals from Palawan, the country’s remaining malaria hotspot, reflecting ongoing exposure and sustained transmission in this region (70). A machine-learning (Random Forest) classification algorithm was developed that enabled strong prediction of recent *P. falciparum* exposure using antibodies to the *P. falciparum* antigens glutamate-rich protein R2 (GLURP R2), early transcribed membrane protein 5 antigen 1 (Etramp5.Ag1), gametocyte-exported protein 18 (GEXP18), and merozoite surface protein 1 19kD (MSP1_19_); however, prediction was sub-optimal for *P. vivax*. We now plan to repeat these analyses using our optimized panel of eight *P. vivax* serological exposure markers (5, 71), with the addition of *P. vivax* AMA1 as a marker of long-term exposure, as highlighted in this work from the Philippines (70). Interestingly, this study in the Philippines (for *P. falciparum*) found the strongest performance when also including age as a covariate (70), whereas our prior work on *P. vivax* did not (5). Beyond *P. falciparum* and *P. vivax*, there has been limited development of well-defined serological panels for other human-infecting *Plasmodium* species, such as *Plasmodium malariae, Plasmodium ovale curtisi, Plasmodium ovale wallikeri,* and *P. knowlesi*. The increasing availability of high-quality genomic data of these less-studied species has now enabled the expression and evaluation of selected corresponding proteins, thereby expanding the antigen repertoire available for serological assays. This advancement will support a more comprehensive assessment of malaria exposure across all *Plasmodium* species using serological methods (72, 73), which requires evaluation of antibody longevity and species-specificity to generate actionable data. As countries progress towards malaria elimination, it will also be important to consider integration of malaria serology with serology for other locally relevant infectious diseases through multi-pathogen and vector (74) serological surveillance (Fig. 1). This will likely improve uptake of such technologies and provide advances towards countries' multi-disease elimination goals.

An important aspect of using serology for surveillance is the timing of sero-surveys and the populations enrolled. Ideal sentinel populations can include school-aged children (64), as they are easy to access operationally at one time, and pregnant women, who report regularly to antenatal clinics (75); however, the latter group may not be ideal for immune-based measures such as sero-surveillance due to the immunoregulatory impact of pregnancy on the immune system (76). Children are also an interesting sub-group for surveillance, as in addition to antibody temporal kinetics guiding definitions of recent versus past exposure, the age of children restricts when they could have had expose to the pathogen of interest. Importantly for our panel of *P. vivax* antigens, serological classification of recent exposure in the prior 9 months has been successful in pediatric cohorts even though overall antibody responses were slightly lower (5). Other opportunistic sampling approaches include piggy-back efforts on nationwide surveys for priority diseases (i.e., national health surveys, malaria indicator surveys) (77), health-facility based surveys that include both treatment-seeking individuals as well as companions (71), or possibly even residual blood donor/bank samples (78). Furthermore, in the context of malaria and in eliminating regions, such as the Asia-Pacific, screening of mobile or migrant populations at borders may be beneficial (79). Each has its own strengths and limitations and needs to be considered within the local healthcare and policy context.

Serological assays may play an increasingly important role to guide targeted interventions in elimination settings. By revealing spatial and temporal patterns of exposure, serological markers can support targeted intervention strategies essential for preventing resurgence (66, 71). Consequently, serology can serve as a critical component of malaria surveillance systems in the final stage of elimination, offering a robust and scalable approach for monitoring ongoing transmission and assessing progress toward malaria-free status (80). They can also be used to directly guide individual-level treatment decisions, such as serological testing and treatment (SeroTAT) (81). SeroTAT aims to provide a valuable alternative to the two main approaches for accelerated malaria elimination campaign-based activities, namely mass screening and treatment (MSAT) and mass drug administration (MDA). For *P. vivax*, MSAT approaches using blood-stage diagnostics are ineffective at reducing *P. vivax* transmission (82), likely as they cannot identify and eliminate the hypnozoite reservoir (limited sensitivity). MDA, when including primaquine treatment to eliminate hypnozoites, is highly effective at reducing *P. vivax* transmission (83) but results in high levels of over-treatment (zero-specificity). SeroTAT aims to provide a balance with substantially improved performance compared with MSAT, and while having lower efficacy than MDA, is predicted to still be effective in reducing *P. vivax* transmission with lower levels of over-treatment (5, 81, 84). Our current *P. vivax* serological assay employs multiplexed Luminex technology to measure total IgG antibody responses to all eight antigens simultaneously in a small sample volume (85), however, there are other laboratory-based methods to measure serological responses, as reviewed recently (7). While all these methods provide high analytical sensitivity, their reliance on laboratory infrastructure limits their utility for real-time surveillance and treatment decisions in remote or resource-constrained settings. Therefore, there is interest in the development of serological point-of-care/contact (POC) tests for *P. vivax* malaria (Fig. 1).

Current POCs, such as RDTs, used in malaria programs detect parasite antigens, such as histidine-rich protein 2 (HRP2) or parasite lactate dehydrogenase (pLDH), enabling identification of active blood-stage *Plasmodium* infections. However, these antigen-based tests have limited sensitivity for *P. vivax*, where infections are frequently asymptomatic and characterized by low-density parasitemia, and they cannot detect individuals with only liver-stage hypnozoites (7). Hence, the innovation required in POC tools is to adapt laboratory-based assays that measure total IgG as biomarkers of recent exposure in the prior 9-months and, by proxy, hypnozoite carriage, to a RDT. Multiple programs of work are now ongoing to enable this reality and support *P. vivax* SeroTAT programs. However, POC serological tools may be restricted to fewer antigens of interest and may be of lower sensitivity, especially if not combined with a semi-quantitative or quantitative reader system. It will thus be essential to evaluate the sensitivity and specificity of novel POC serological tools developed and to consider these against serology use-cases to determine whether their benefits (reaching remote populations, easy-to-use) outweigh any drops in performance. Beyond serology, broader innovations in malaria POC antigen diagnostics aim to improve sensitivity, expand species detection, and integrate molecular and immunological technologies into field-ready platforms. Although conventional RDTs remain widely used, their performance is constrained by antigen variability, including the emergence of *P. falciparum* parasites with HRP2/HRP3 gene deletions that compromise test accuracy (86). In response, next-generation RDTs with improved antigen targets and multiplex detection capabilities are under development. Parallel advances in molecular POC technologies, such as loop-mediated isothermal amplification (LAMP) and microfluidic PCR-based devices, seek to deliver laboratory-level sensitivity in a portable system suitable for remote settings (87, 88). Collectively, these innovations reflect a broader shift toward highly sensitive, field-deployable diagnostic tools that can strengthen surveillance as intervention strategies, guide targeted responses, and support health systems in the stage of malaria elimination. The convergence of serological, molecular, and antigen-based POC technologies holds significant promise for accelerating progress toward malaria-free status in endemic countries, with clear translational potential to other infectious diseases. In addition to improved diagnostics and surveillance capabilities, along with novel interventions, the development of vaccines or immune-based therapeutics for malaria may be important for sustaining gains and elimination long-term. Importantly, serology can also play a valuable role in characterizing protective immunity and guiding vaccine design.

## MEASURING FUNCTIONAL ANTIBODY RESPONSES AT HIGH-THROUGHPUT

Antibodies are a vital mediator of protection and pathogen control from infectious diseases. In contrast to serology for surveillance of exposure, serology for understanding protective immunity requires measurement of antibody function and not just magnitude. In the context of malaria, parasite-specific antibodies can play a critical role in protective immunity via multiple mechanisms, as previously reviewed (89, 90). Beyond direct inhibition or neutralization, antibodies can mediate the destruction of the pathogen or infected cells via complement fixation and activation or through interactions with Fcγ-receptors, leading to downstream effector mechanisms, such as complement-dependent cytotoxicity (CDC), antibody-dependent cellular phagocytosis (ADCP), and antibody-dependent cellular cytotoxicity (ADCC) (89, 90). A range of medium to high-throughput *in vitro* assays have been developed to quantify antibody effector functions, such as antibody inhibition of receptor-binding assays using quantitative or competitive ELISA (91, 92), growth inhibition assays (GIAs) to assess antibody-mediated effects on parasite growth (93), and multiplex assays measuring antibody-driven Fcγ-receptor binding and complement fixation (6). A plate-based flow cytometric assay using THP-1 monocytic cell lines expressing Fc receptors, along with the use of antigen-coated fluorescent beads, has been used to measure opsonic phagocytosis instead of using parasite cultures (94). PBMC-based *in vitro* assays have been used to assess natural killer (NK) cell ADCC activity by measuring degranulation and IFNγ production (95). Our understanding of antibody function and the development of assays to test the functionality in malaria mostly come from research on *P. falciparum*, partially due to challenges in maintaining long-term *in vitro* cultures of *P. vivax* parasites (96). A systems serology approach can be used to overcome this challenge, through a focus on antigen-specific responses rather than the use of whole parasites, and by measuring engagement of antigen-specific antibodies with Fcγ-receptors or complement proteins as markers of downstream effector functions (Fig. 2). This approach can enable identification of correlates of protection (97) and can guide subsequent resource-intensive assays like GIA or cell-based assays that, for *P. vivax,* may need to be conducted in endemic areas with access to patients as a source of parasite material.

**Fig 3:**
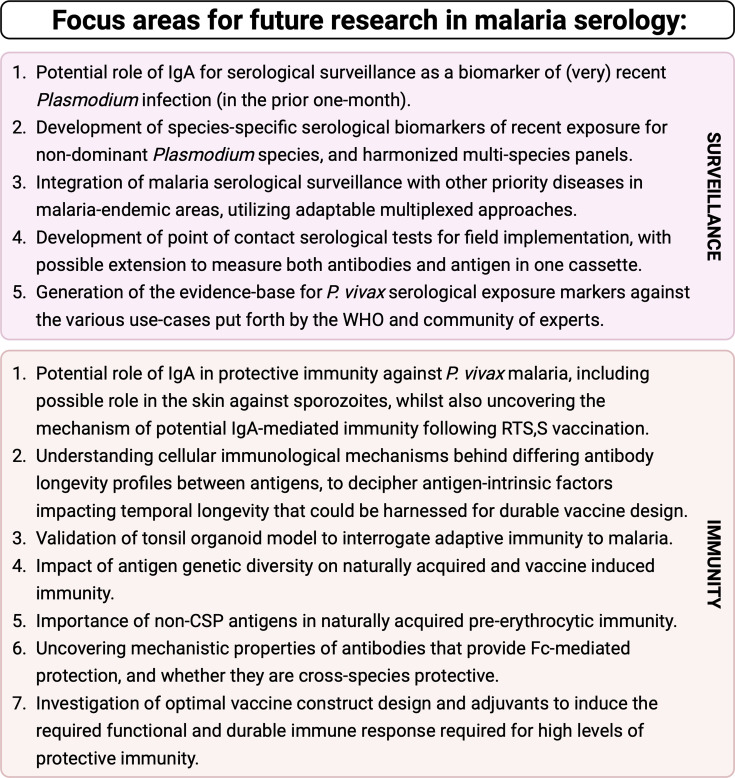
Focus areas for future research in malaria serology across the surveillance and immunity fields.

In the context of *P. falciparum* malaria, the system serology approach has been used to characterize functional responses to malaria vaccination and to identify correlates of protection against clinical malaria, cerebral malaria, and placental malaria. RTS,S/AS01 (herein referred to as RTS,S) was the first licensed malaria vaccine recommended by the WHO, and targets the major sporozoite coating antigen, circumsporozoite antigen (CSP). This vaccine has moderate efficacy (31.3%–55.8% in the first 12 months in infants and children [98]) and limited durability, and therefore efforts have been taken to assess the role of functional antibodies in RTS,S vaccination across multiple studies to define correlates of protection to enable the generation of more effective second-generation vaccines. Systems serology approaches have identified various potential correlates of protection, including engagement of FcγRIIa/III and downstream neutrophil, monocyte, and THP-1 phagocytosis (99) (100), as well as NK cell-mediated ADCC (100). These functional responses have peaked following each vaccine dose and declined within a year, corresponding to an observed reduction in vaccine efficacy. IgA responses have also recently been associated with protection induced by RTS,S (101), as well as the experimental *P. falciparum* vaccine targeting the blood-stage antigen reticulocyte-binding protein homolog 5 (Rh5) (102). Monomeric IgA possibly provides protection through its ability to bind FcαRI and promote downstream opsonic phagocytosis. A recent study was able to demonstrate improved neutrophil functional activity against *P. falciparum* merozoites by modifying an isolated mAb to an IgG-IgA bi-isotype mAb (103). In humans, IgA has two subclasses, IgA1 and IgA2, and these have been shown to be differentially glycosylated, with IgA2 inducing greater effector functions (104). Interestingly, in naturally exposed and protected individuals from Mali, several IgA monoclonal antibodies were isolated that targeted a conserved epitope within CSP (105). Sporozoites spend most of their extracellular time at the skin inoculation site before entering the bloodstream, making the skin one of the most vulnerable sites for antibody-mediated destruction (106). However, there are relatively few studies on mechanisms of immunity at the skin compared with blood and liver stages. It seems plausible to develop strategies inducing skin-resident IgA (107) to achieve sterile protection by blocking the parasite at its earliest encounter with the host, and this warrants further investigation and innovation in the use of models of skin immunity, as has been seen for other pathogens (108, 109) (Fig. 3).

Other studies in the context of naturally acquired immunity to *P. falciparum*, rather than vaccination, have focused on using systems serology to uncover antibody features associated with protection from various forms of severe malaria. A recent study carried out in Malawian children with cerebral or uncomplicated malaria was able to differentiate these two groups by functional features of antibody responses (110). Notably, antibodies against the *P. falciparum* erythrocyte membrane protein 1 (PfEMP1) that bound C1q and FcγRIIIb were identified as key mediators of protection against cerebral malaria in children, primarily by promoting parasite clearance through antibody-dependent neutrophil phagocytosis (ADNP) (110). Similarly, in a cohort of 77 pregnant women from Papua New Guinea (PNG), a systems serology approach characterizing antibody responses to the *P. falciparum* protein VAR2CSA, that binds to Chondroitin Sulfate A in the human placenta, was able to identify mid-pregnancy antibody features associated with protection from placental malaria at delivery (111). In the context of *P. vivax* malaria, we recently conducted the first extensive study on functional correlates of naturally acquired immunity. Our findings from a longitudinal cohort of young children from PNG, naturally exposed to *P. vivax*, identified antigens targeted by multi-functional antibodies (multi FcγR-binding and/or complement fixing) as well as combinations of two or three antigens strongly associated with protection against clinical malaria (6). Specific antigens are discussed further below. While there is no licensed vaccine against *P. vivax,* one of the most advanced candidates is the antigen DBPII, a key invasion ligand that mediates entry into reticulocytes. In a *P. vivax* DBPII vaccine challenge trial, complement C1q and Fcγ receptors (FcγRIIa/III) were identified as the functional features most strongly associated with reduced parasite multiplication rate, thereby defining a functional antibody signature of protection (112). Taken together, these studies illustrate the power of systems serology to comprehensively characterize functional antibody responses across different contexts of malaria, providing new insights into correlates of protection of existing vaccines and identifying new target antigens and mechanisms of protection to be taken forward for next-generation vaccines.

## USING SEROLOGICAL INSIGHTS OF PROTECTIVE IMMUNITY TO DESIGN BETTER VACCINES

Insights into protective immune responses derived from systems serology and downstream mechanistic assays can be used to validate novel targets of protective immunity, using data from sero-epidemiological studies of naturally acquired immunity or through access to CHMI samples (Fig. 2). Furthermore, such findings can be used in the design of more effective vaccines for malaria and other infectious diseases. While CHMI studies are powerful as variables can be controlled, i.e., timing of infection, prior exposure, etc, they have only been conducted in adults (113), and thus valuable insights on target populations including children can still be gained from studies of naturally acquired immunity. For *P. vivax*, traditional methods to elucidate essential proteins, such as conditional gene knockouts in parasite and animal models, are difficult due to the lack of an *in vitro* culture system. The 10 *P. vivax* vaccines in clinical development target just four antigens, CSP, DBPII, and two transmission-blocking targets Pvs25 and Pvs230, alongside one whole-sporozoite approach (114). Endemic cohort studies combined with a systems serology approach are a useful tool to discover potential vaccine candidates. Multiplexed technology allows for the high-throughput screening of antigens for their associations with protection against clinical disease, which can aid in the discovery of novel vaccine targets. Indeed, an early multiplex serology study with a panel of 38 antigens demonstrated that total IgG antibodies to biologically relevant parasite antigens like erythrocyte-binding protein II (EBPII) and cysteine-rich protective antigen (CyRPA), a vaccine candidate for *P. falciparum* malaria (115), as well as the StAR-related lipid transfer protein (StART [PVX_081550]), show better correlation against protection of *P. vivax* clinical disease in children from PNG compared with DBPII (116). We have further tested a larger panel of 342 *P. vivax* proteins using a different high-throughput methodology (AlphaScreen assay), which validated StART as one of the top hits from this same PNG cohort (117), and warrants further investigation. EBPII has been reported to be important for parasite binding to the reticulocyte surface (118, 119), and CyRPA and StART have essential roles for parasite survival in *P. falciparum* with potential conserved function in *P. vivax* (120–122). These findings highlight how down-selection of proteins through high-throughput assays can identify novel vaccine targets that may be more efficacious than current clinical candidates. In *P. falciparum,* systems serology has highlighted that the breadth of antibody-Fc mediated effector functions strongly determines clinical immunity (123), which we have further shown to be relevant for *P. vivax* infections (6), providing novel data not only on which antigens to target but also the effector mechanism we should be aiming to induce through vaccination. Here, through screening functional antibody responses to a panel of 30 *P. vivax* proteins, we identified key targets such as the Pv-fam-a family of proteins, MSP3α, and RON2 for Fc engagement and MSP5 for complement fixation (6). Further research is ongoing exploring downstream effector functions, determining cross-species protective immunity, and expanding the panel to >70 *P*. *vivax* antigens, including pre-erythrocytic targets (where the focus has, to date, been nearly entirely on CSP for *P. vivax*). Expansive panels are a key benefit of multiplexed technology and are also relevant for assessing the impact of antigen genetic diversity, as we have done in the context of serological surveillance (61). Variant proteins can be expressed based on known diversity of candidate vaccine antigens, and sero-epidemiological studies assessing associations with protection (both antibody magnitude and function) could be compared. For example, the most advanced *P. vivax* blood-stage vaccine candidate, DBPII, has high global nucleotide and haplotype diversity, but studies so far point to limited impact for both antibody binding (61) and function (112). Several other *P. vivax* vaccine candidates in the pre-clinical pipeline also exhibit high levels of genetic diversity (124) and require further research to understand the potential impact on protective immunity (Fig. 3).

Malaria vaccine research has mostly focused on the ability to generate nAbs that prevent parasite function; however, future vaccines should consider antigenic targets, adjuvants, or vaccine platforms that can induce nAbs with strong Fc-mediated effector functions. This has been particularly important for SARS-CoV-2 vaccines, where multiple studies have identified that repeated monovalent and bivalent mRNA booster doses lead to increased levels of anti-Spike IgG2 and IgG4 compared with protein-based vaccines (125–128), and antigen-specific IgG4^+^ B-cells compared with heterologous prime-boost vaccines (adenovirus/mRNA versus mRNA/mRNA) (129, 130). IgG4 and IgG2 have impaired binding to activating Fcγ-receptors compared with IgG1 and IgG3 (131, 132), and indeed increased IgG4 levels have been negatively correlated with Fcγ-receptor engagement (125). High IgG4 levels have been found to impair ADCP and ADCC through competition with other IgG subclasses to Fcγ-receptors but may bind synergistically to FcγRI and FcγRIIIa with other subclasses when total antigen-IgG levels are low (131, 133). These findings from systems serology demonstrate that future COVID-19 vaccines should consider the type of booster vaccines used to induce higher levels of cytophilic IgG subclasses and reduced serum IgG4 levels and highlight more broadly the importance of defining beneficial functional correlates early in vaccine design. As mentioned earlier, and notably outside the context of a pandemic, challenge trials, such as CHMI, can be utilized to deeply characterize immunity to both natural infection and candidate vaccines. Multiple homologous *P. vivax* CHMI rechallenges (in malaria-naïve individuals) have found that a single *P. vivax* CHMI is sufficient in inducing long-lived clinical immunity (134). Individuals who had primary versus secondary CHMI had similar reductions in parasite multiplication rate, supporting the notion that anti-parasite immunity is acquired slowly after many years of repeated exposure to malaria. Furthermore, heterologous challenge with *P. falciparum* demonstrated that clinical immunity is species-specific, as *P. vivax* CHMI was not protective against *P. falciparum* (134)*,* although, to our knowledge, this has yet to be tested for the more closely related parasite *P. knowlesi*. CHMI studies have primarily been undertaken in high-income countries despite malaria burden and endemicity occurring in lower- and middle-income countries (135). Future CHMI studies, such as the Malaria Infection Study in Thailand (MIST), will provide an experimental clinical platform to test the efficacy of future vaccine and antimalarial drug candidates against *P. vivax* in a region where the disease is endemic (136). CHMI studies following vaccination with potential candidates have already been undertaken, e.g., PvDBPII, PvCSP (137), and radiation-attenuated sporozoites (RASs) (138). These provide opportunities for identification of both correlates of protection and protective monoclonal antibodies, which can bring further precision to vaccine design (139), or the latter can be used as a treatment itself (140).

Durable and specific vaccine-induced immune responses can be driven by a range of factors considered during vaccine design. While nAbs induced by PfCSP vaccines are important for preventing sporozoite traversal and invasion of hepatocytes (141), CD8^+^ T cells through their ability to kill infected hepatocytes also play a critical role in vaccine-mediated liver-stage immunity (142). Indeed, liver-resident memory CD8^+^ T cells (Trm), which can rapidly respond upon re-infection, are essential for protection in radiation-attenuated sporozoite (RAS) vaccinated mice (143), with indirect evidence also in humans (144). Although mRNA vaccines can elicit circulating memory CD8^+^ T cells (effector memory and central memory), the liver Trm cells, which are critical for protection, are inefficiently induced. However, chemical modifications to a natural killer T-cell agonist, α-galactosylceramide, when included as an mRNA vaccine adjuvant, were able to help induce high levels of peptide-specific Trm cells, which strongly correlated with sterile immunity (145). This study was performed in mice with *P. berghei* but could potentially help drive longer-lived pre-erythrocytic vaccine efficacy in humans. No head-to-head comparisons have been performed against RTS,S/AS01 and R21/Matrix-M so far in endemic countries (146, 147), but future studies comparing whether the higher density of PfCSP on vaccine scaffolds and different adjuvants influence vaccine efficacy and durability would aid in improved vaccine design. The repetitive and multimeric antigen display, e.g., virus-like particles used in HPV and current malaria vaccines, generates more durable immune responses compared with monomeric protein or polysaccharide vaccines (148). For HPV vaccines, the densely ordered and repetitive display of B cell epitopes on a particulate scaffold help with cross-linking of BCRs on naïve B cells, leading to better germinal center interactions, which generate greater activation and survival signals (149). This generates higher frequencies of LLPCs, which continuously secrete nAbs to prevent HPV entry into cells. Dosing regimens can also be critical in inducing preferred vaccine responses. This has been exemplified in the context of malaria and COVID-19 vaccines. Delayed boosting by 4–6 months may enable germinal center interactions, allowing for affinity maturation, isotype switching, and memory B cell generation (150, 151). RTS,S (152), PfRh5 (102), and some SARS-CoV-2 mRNA (153) and viral vector (154) vaccines performed better with delayed dosing in naïve individuals. For *P. vivax* infections, a delayed dosing regimen of protein-based PvDBPII-Matrix-M vaccine showed higher IgG antibody levels and was better at reducing parasite multiplication rate (155). The PvDBPII Matrix-M and PvDBPII viral vector vaccine were further compared, and higher levels of SalI-strain specific IgG and inhibition of DBPII to its receptor DARC were noted for the Matrix-M group (155). Antibodies that block DBPII binding to DARC are the key mechanism behind DBPII-targeted parasite growth inhibition (92). Overall, these studies provide insights into how we can utilize new technologies like adjuvants and mRNA vaccines to help drive effective *P. vivax* vaccine design in the future and highlight that knowledge on protective immunity gained from sero-epidemiological or CHMI studies can be implemented and targeted with clever vaccine design (Fig. 2).

## CONCLUSIONS

Serological techniques can provide unique insights into the immune response to malaria and other infectious diseases, which can be leveraged in numerous ways—including for surveillance approaches and to design optimal vaccines, as discussed here. To provide actionable data, studies need to be designed carefully. For serological surveillance, an understanding of temporal antibody longevity is essential for biomarkers to inform on recent exposure to the pathogen, and not just overall lifetime exposure. For understanding protective immunity and to guide vaccine design, studies need to measure antibody function and not just antibody magnitude. While much exciting research has already been undertaken in this space, there are key areas we see for future research (Fig. 3). Beyond this, to ensure the best chance of translation and implementation of novel approaches built on malaria serology, researchers need to engage with malaria-endemic communities and stakeholders to ensure feasibility and identify any barriers to successful up-take.
